# Fine-needle aspiration of axillary lymph nodes: a change of paradigm in the approach to axillary compromise?

**DOI:** 10.1590/0100-3984.2016.49.1e3

**Published:** 2016

**Authors:** Linei A. B. D. Urban

**Affiliations:** 1 Responsible for the Breast Unit at Clínica DAPI - Diagnóstico Avançado por Imagem, Curitiba, PR, Brazil. Coordinator of the Mammography Commission - Colégio Brasileiro de Radiologia e Diagnóstico por Imagem (CBR). E-mail: lineiurban@hotmail.com.

Breast cancer is the most common malignant tumor in women. Recently, the Brazilian radiological literature has been extremely concerned with the relevance of the role played by imaging methods in the improvement of breast cancer diagnosis^(1,6)^. In the last decades, the treatment for breast cancer has undergone major changes, with more conservative surgeries demonstrating no influence on overall survival. However, despite the recent developments, axillary compromise still remains as the most relevant isolated prognostic factor. Additionally, it may determine the indication for the most appropriate treatment such as chemotherapy and radiotherapy in a significant number of patients.

Until recently, sentinel lymph node biopsy has been a game changer in the assessment of the axilla and definition of the necessity of axillary dissection. The latter, however, was responsible for the major part of the morbidity associated with breast cancer surgery. Avoiding such a measure in cases where it is unnecessary, i.e. in cases of negative sentinel lymph node was the main focus of the oncologic breast surgery over the last decades.

Thus, the application of a minimally invasive, simple and effective diagnostic method in the prediction of axillary compromise could be helpful to reduce the surgical time, so the surgeon could avoid the sentinel lymph node biopsy, proceeding directly to axillary dissection. Would it be so simple? Such a rationale would be perfect up to four years ago, before the publication of the clinical essays ACOSOG Z0011^(7)^ and AMAROS^(8)^. Such studies have demonstrated that patients eligible for conservative surgery (T1-T2), with less than three positive lymph nodes and submitted to axillary dissection, presented the same survival and local management as those who had not undergone dissection.

Then, on the basis of the ACOSOG Z0011 and AMAROS results, in which situations could the findings by Rocha et al.^(9)^ be useful?

The great relevance of the study developed by Rocha et al. is in the high degree of sensitivity of fine needle aspiration biopsy (FNAB) observed in cases of lymph nodes considered to be suspicious and indeterminate (87.1%), allowing to avoid the sentinel lymph node procedure in 70.1% of patients. And, in the cases where FNAB was negative, could also sentinel lymph node biopsy be avoided? The answer is still no. The negative predictive value was 69.5%, similar to the value found in other studies. On the other hand, in cases where the lymph node was considered to be normal at ultrasonography, FNAB also did not add any useful information since the lymph node selected for puncture might not be the breast drainage "sentinel" lymph node.

Thus, despite the relevant changes in the approach to the axilla, ultrasonography-guided axillary FNAB still has its place in the clinical practice. It may be indicated in cases of invasive breast cancer with altered lymph nodes at ultrasonography, independently from the tumor size and histological type.

The challenge for the future is to demonstrate that the axillary lymph node positiveness found at FNAB in the study developed by Rocha et al. can compare to the sentinel lymph node positiveness in patients assessed in the ACOSOG Z0011 and AMAROS. In other words, to demonstrate that patients with a positive FNAB, but with an early-stage tumor (T1-T2) and clinically negative axilla can dispense with axillary dissection in the future. This clearly demonstrates that the role played by the XXI century radiologist is changing. We must increasingly take our responsibility in the multidisciplinary evaluation of diseases, particularly in cases of breast cancer.
